# Cross-Talk Between Tumor Cells and Stellate Cells Promotes Oncolytic VSV Activity in Intrahepatic Cholangiocarcinoma

**DOI:** 10.3390/cancers17030514

**Published:** 2025-02-04

**Authors:** Victoria Neumeyer, Purva Chavan, Katja Steiger, Oliver Ebert, Jennifer Altomonte

**Affiliations:** 1 Department of Internal Medicine 2, University Hospital of the Technical University of Munich, 81675 Munich, Germany; 2 Department of Pathology, Technical University of Munich, 81675 Munich, Germany

**Keywords:** oncolytic virus, cholangiocarcinoma, cancer, fibrosis, hepatic stellate cells, intratumoral fibroblasts

## Abstract

As the mechanisms underlying tumorigenesis become better understood, the dynamic roles of cellular components of the tumor microenvironment, and their cross-talk with tumor cells, have come to light as key drivers of disease progression and have emerged as important targets of new cancer therapies. In the field of oncolytic virus (OV) therapy, stromal cells have been considered as potential barriers to viral spread, thus limiting virus replication and therapeutic outcome. However, new evidence indicates that intratumoral fibroblasts could support virus replication. We have demonstrated in a rat model of stromal-rich intrahepatic cholangiocarcinoma (CCA) that vesicular stomatitis virus (VSV) can be localized within intratumoral hepatic stellate cells (HSCs), in addition to tumor cells, when the virus was applied via hepatic arterial infusion. Furthermore, VSV was shown to efficiently kill CCA cells and activated HSCs, and co-culture of CCA and HSCs increased viral titers. Interestingly, this effect is also observed when each cell type is cultured alone in a conditioned medium of the other cell type, indicating that secreted cell factors are at least partially responsible for this phenomenon. Partial reduction in sensitivity to type I interferons was observed in co-culture systems, providing a possible mechanism for the increased viral titers. Together, the results indicate that targeting activated HSCs with VSV could provide an additional mechanism of OV therapy, which, until now has not been considered. Furthermore, these findings suggest that VSV is a potentially powerful therapeutic agent for stromal-rich tumors, such as CCA and pancreatic cancer, both of which are very difficult to treat with conventional therapy and have a very poor prognosis.

## Simple Summary

Cholangiocarcinoma is a tumor type characterized by a dense and fibrotic microenvironment. Therefore, it is difficult to treat, and patients often face limited therapy options and poor prognosis. Oncolytic viruses are viruses that specifically kill cancer cells and offer a potential alternative therapy for several cancer types. The aim of this study was to analyze the potential of oncolytic vesicular stomatitis virus (VSV) as a therapy for fibrotic cholangiocarcinoma. We confirmed that treatment with VSV was able to efficiently kill cholangiocarcinoma cells and reduce tumor fibrosis in an animal model of intrahepatic cholangiocarcinoma. Thus, treatment with VSV could have therapeutic potential in difficult-to-treat fibrotic cancer types such as cholangiocarcinoma or pancreatic cancer.

## 1. Introduction

Cholangiocarcinoma (CCA) is the second most common primary hepatic cancer with a globally rising incidence [1,2]. Surgical resection is the only curative therapy, but due to the late stage at which it is often diagnosed, patients often present with unresectable tumors, leading to high recurrence rates [3] and a poor 5-year survival rate of 5% [4]. Interestingly, tumor progression and invasion of CCA have been strictly linked to desmoplasia produced by recruited cancer-associated fibroblasts (CAFs) [5,6], and overall survival, as well as progression-free survival, were shown to be higher in patients presenting with low-grade fibrosis than those with advanced fibrosis [7]. Additionally, activated hepatic stellate cells (HSCs), which are the main precursor of CAFs in the liver [8,9,10], were shown to promote CCA progression in vitro and in vivo [11]. Thus, high levels of alpha-smooth muscle actin (α-SMA) expressed by CAFs derived from HSCs correlated with poor prognosis [12], and these CAFs were responsible for desmoplasia in the context of CCA [13,14,15,16,17,18]. The fibrotic tumor microenvironment (TME) was shown to facilitate tumor progression [19] and to support resistance to chemotherapy [20], further highlighting the important role of CAFs in CCA. On a molecular level, HSCs were shown to be activated by TGF-β, which is secreted by cancer cells [21,22,23,24] and was found to be overexpressed in CCA stroma and to correlate with poor prognosis [25]. In turn, activated HSCs were shown to secrete several cyto- and chemokines, such as TNFα, GM-CSF, IL-6, IL-10, and TIMP1, which induce acute inflammation and fibrosis in the liver, further altering the TME [21].

Vesicular stomatitis virus (VSV) has been extensively explored as an oncolytic agent and has shown promising therapeutic effects in multiple solid tumor indications, such as hepatocellular carcinoma (HCC) [26,27,28], melanoma [29], and pancreatic ductal adenocarcinoma (PDAC) [30]. In a previous study, we have shown that VSV was able to specifically replicate in activated HSCs and to reduce αSMA levels in these cells. Furthermore, VSV was detected in HSCs present in hepatic fibrosis in a rat model of chemically-induced HCC, and virus treatment significantly improved liver staging and reduced intrahepatic collagen content and αSMA levels [31]. In that study, VSV induced apoptosis in HSCs in vivo, which was shown to be one of the major mechanisms for the regression of fibrosis [32,33]. In line with that, another OV, Newcastle disease virus (NDV), was shown to selectively replicate in activated but not quiescent HSCs in vitro and in vivo. NDV infection led to the downregulation of αSMA, TIMP, and collagen and induced apoptosis of HSCs. In a murine model of liver fibrosis, treatment with NDV led to a drastic reduction of fibrosis [34], further highlighting the potential of OVs to alter tissue architecture. Additionally, CAFs were shown to be sensitized to OV infection by cancer cell-conditioned medium or TGF-β, suggesting an important role in the crosstalk between CAFs and cancer cells during OV treatment [35]. In that study, increased replication of a VSVΔM51 variant was observed in co-culture or conditioned media experiments using renal carcinoma, PDAC, or ovarian cancer cells, and OV therapy was enhanced in vivo in a xenograft model when cancer cells were injected together with CAFs compared to cancer cell injection alone.

Taken together, OV treatment has been shown to not only target tumor cells but also have the potential to alter the tumor microenvironment and to resolve fibrosis surrounding the tumor cells. While these previous studies suggest that OVs, and in particular VSV, may broadly replicate in CAFs and HSCs, this approach had not yet been explored, to our knowledge, for its potential for targeting intratumoral HSCs and modulating the cross-talk between cholangiocarcinoma cells and HSCs, thereby providing an additional and yet unexplored mechanism of action in this aggressive, stromal-rich malignancy. Here, we demonstrate that VSV is able to replicate in activated HSCs as well as in CCA cells in vitro, and crosstalk between cancer cells and fibroblasts could enhance viral infection by dampening the antiviral interferon (IFN) response. In a rat model of fibrotic CCA, VSV was detected in both CCA cells and intratumoral HSCs, and treatment was shown to reduce the gene expression of TGF-β and αSMA and to reduce the fibrotic content of these lesions. Thus, due to its ability to target tumor cells and CAFs and its antifibrotic characteristics, oncolytic VSV is well suited for the treatment of stromal-rich tumors, such as CCA.

## 2. Materials and Methods

### 2.1. Cell Culture, Co-Culture, and Conditioned Media Experiments

Human CCA cell lines, HuCCT1 and RBE, were cultured in RPMI medium (Invitrogen, Carlsbad, CA, USA) supplemented with 10% fetal calf serum. LX-2 cells and primary human HSCs were cultured in DMEM GlutaMAX™-I (Invitrogen) supplemented with 2% FCS, and 10% FCS, respectively. BHK-21 cells were cultured in GMEM BHK-21 medium (Invitrogen) supplemented with 10% FCS and 2% tryptose phosphate broth. For co-culture experiments, CCA cells were mixed with HSCs at a ratio of 1:1, and co-culture was allowed to establish for 48 h prior to infection with VSV. For conditioned media experiments, CCA cells and HSCs were plated in 6-well plates and cultured in DMEM GlutaMAX™-I containing 5% FBS for 48 h. The medium was collected, centrifuged, and sterile-filtered. CCA and HSCs were preconditioned in the respective medium for 48 h prior to further usage.

### 2.2. Virus Production

Virus stocks were produced in adherent BHK-21 cells cultured in 15 cm dishes. BHK-21 cells were infected at a multiplicity of infection (MOI) of 0.0001 for 48 h. Virus-containing supernatant was collected and cleared from cell debris by centrifugation at 500× *g* using a benchtop centrifuge, followed by ultracentrifugation using a type 70 Ti fixed angle rotor at 24,000 rpm for 1 h. Virus pellets were resuspended in PBS and purified over a sucrose gradient ranging from 10% to 60% sucrose.

### 2.3. Quantitative Real-Time PCR

RNA was extracted from cells or snap-frozen tissue using an RNeasy Mini Kit (Qiagen, Valencia, CA, USA) and reverse transcribed with Quantitect Reverse Transcriptase (Qiagen). mRNA expression was analyzed using quantitative real-time PCR LightCycler 480 (Roche, Basel, Switzerland) and the KAPA SYBR Fast LightCycler 480 Kit (Roche). Primers are listed in Table 1. Expression levels of the genes of interest were normalized to GAPDH.

### 2.4. Growth Curves

All viral infections were carried out at an MOI of 0.01 unless stated otherwise. To analyze the replication dynamics of VSV, cells were plated in 24-well plates at a density of 10^5^ cells per well and infected with VSV-GFP in PBS containing Mg^2+^ and Ca^2+^ for 1 h. Cells were then washed to remove free viral particles and cultured in their respective medium. Samples for viral titer analysis were collected from the supernatant at the indicated time points. Viral titers were determined using the 50% tissue culture infective dose (TCID_50_) assay in BHK-21 cells.

### 2.5. Cytotoxicity Assay

Cytotoxicity caused by VSV infection was measured by assessing the release of lactate dehydrogenase (LDH) into the supernatant. Supernatants from infected cells were collected, and LDH was measured using the CytoTox 96 Non-Radioactive Cytotoxicity Assay (Promega, Madison, WI, USA), and absorption was measured at 450 nm. A maximum release control was obtained by lysing one well of uninfected cells at each time point, and data was plotted as the percentage of maximum cytotoxicity.

### 2.6. Viability Assay

Cell viability measurements were based on the reduction of MTS compounds using the CellTiter96 AQueous One Solution Cell Proliferation Assay (Promega). Briefly, MTS was added to the cells and incubated at 37 °C according to the manufacturer’s instructions. Absorption was measured at 490 nm. Values were normalized to untreated controls at each time point and are plotted as percent of total viable cells.

### 2.7. IFN and ISRE Reporter Assays

Promoter activation of the IFN gene and the interferon-stimulated response element (ISRE) were analyzed by a luciferase reporter assay. Briefly, cells were transfected with 100 ng of p125 plasmid (Promega) expressing firefly luciferase under the control of the IFNβ promoter and of pISRE plasmid (Promega) expressing firefly luciferase under the control of interferon-sensitive response elements (ISRE), respectively. A total of 10 ng of pRL plasmid (Promega), constitutively expressing Renilla luciferase, were co-transfected into each well as a transfection control. After 24 h, cells were either mock-treated, or stimulated with poly I:C (2.5 μg/mL), universal type I IFN (500 IU), VSV-GFP, or VSV(M51R)-GFP at an MOI of 1 and incubated overnight. Luciferase activity was then measured using the Dual-Luciferase Reporter Assay kit (Promega) according to the manufacturer’s instructions. Firefly luciferase activity was normalized to Renilla luciferase, and data were expressed as fold-change compared to mock-treated control.

### 2.8. IFN Protection Assay

IFN protection assay was used to determine the responsiveness of CCA cells and HSCs to IFN stimulation in the context of VSV infection. For this, cells were pretreated overnight with increasing concentrations of Universal type I IFN (PBL Interferon Source, Piscataway, NJ USA), followed by VSV infection at an MOI of 1 in the continued presence of IFN. Supernatants from infected cells were collected at 24 h post-infection, and viral titers were determined by TCID_50_ assay.

### 2.9. Animal Experiments

All animal experiments were performed according to the guidelines of the institute’s Center for Preclinical Research, and the Regional Commission for Animal Protection (Regierung von Oberbayern, Munich, Germany), approved under file #55.2-1-54-2531-10.06. Six-week-old male Buffalo rats (Harlan Winkelmann, Borchen, Germany) received a 0.01% solution of thioacetamide or normal drinking water as a control continuously for 19 weeks. Animals with spontaneously occurring cholangiocarcinoma lesions were laparotomized and treated with 10^7^ pfu of VSV-LacZ or PBS in a 1 mL volume via hepatic artery infusion, and animals were sacrificed 1 day after treatment. The tissue was either snap-frozen for RNA extraction and TCID_50_ analysis or fixed overnight in 4% paraformaldehyde for histology.

### 2.10. Histology, Immunohistochemistry, and Immunofluorescence

Formalin-fixed tissue was dehydrated and embedded with paraffin. Paraffin sections of 3 µm in thickness were stained for general histological analysis using hematoxylin-eosin (HE) staining or for analysis of fibrosis using Elastica van Gieson staining according to standard protocols. The degree of fibrosis was quantified after slide digitalization (Aperio CS2, Leica, Wetzlar, Germany) using the Aperio positive pixel count algorithm (red pixels) after annotation of the neoplasms by an experienced Comparative Pathologist (KS). Additionally, immunohistochemistry was performed to analyze the localization of VSV using a VSV-M antibody (Kerafast, Shirley, MA, USA). Immunofluorescence was performed to analyze infection of HSCs using VSV-M antibody and activation of HSCs using an antibody directed against αSMA (Sigma-Aldrich, St. Louis, MO, USA) in conjunction with FITC-conjugated anti-mouse and Cy3-conjugated anti-rabbit antibodies (Jackson ImmunoResearch Laboratories, West Grove, PA, USA).

### 2.11. Statistical Analysis

Data was analyzed and plotted using GraphPad Prism software (GraphPad Software, San Diego, CA, USA). Statistical analysis was performed by applying a Student’s *t*-test, and statistical significance was defined as *p* < 0.05.

## 3. Results

### 3.1. Crosstalk Between CCA Cells and HSCs Induces Pro-Tumorigenic and Pro-Fibrotic Signaling

**Figure 1:**
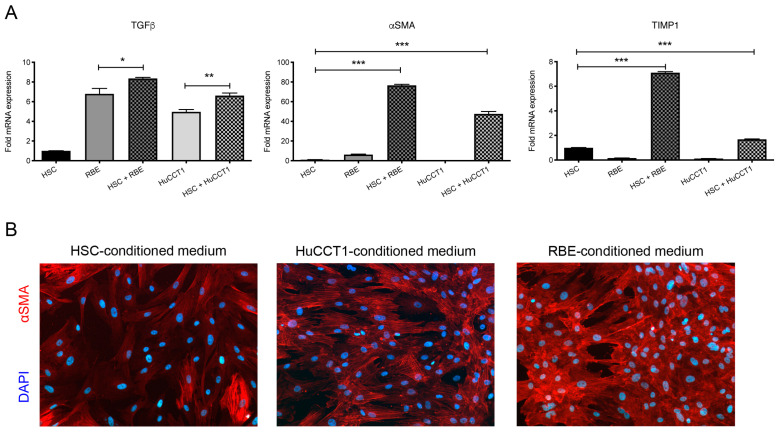
Crosstalk between CCA cells and HSCs leads to differential gene expression and HSC activation. (**A**) Human CCA cell lines, RBE and HuCCT1, and primary human hepatic stellate cells (HSCs) were either cultured alone or as co-culture at a ratio of 1:1. Expression of TGF-β, αSMA, and TIMP-1 were analyzed by quantitative real-time RT-PCR and normalized to GAPDH. Mean values from three independent experiments are shown, and error bars indicate SEM. Statistical significance was determined by Student’s *t*-test (* *p* < 0.05, ** *p* < 0.01, *** *p* < 0.001). (**B**) Primary human HSCs were cultured in their own medium or medium conditioned by RBE or HuCCT1 cells for 48 h. Immunofluorescence staining for αSMA (red) and nuclei (DAPI, blue) was performed. Pictures were taken with a fluorescence microscope at a magnification of 200×.

Crosstalk between HSCs and HCC cells has been studied extensively; however, there is less data available on the interaction between HSCs and CCA cells. To determine, whether CCA cells and HSCs would also have gene modulatory activity on each other, the human CCA cell lines, HuCCT1 and RBE, were co-cultured with primary human HSCs, and gene expression was analyzed. Indeed, upregulation of TGF-β was observed when HuCCT1 and RBE cells were co-cultured with HSCs, while αSMA and TIMP1 were upregulated when HSCs were co-cultured with CCA cells compared to the single culture conditions (Figure 1A). Additionally, HSCs were stained for αSMA by immunofluorescence to analyze their activation status. We additionally conducted studies in which HSCs were cultured in a conditioned medium of either CCA cell line, in order to characterize the effects of one-directional communication of tumor cells on the HSCs. While HSCs cultured in their own medium remained in a rather inactivated state, conditioned medium from CCA cells led to the activation of HSCs as determined by enhanced αSMA expression (Figure 1B). These results indicate that bi-directional crosstalk between human CCA cells and HSCs can lead to mutual activation and induce pro-tumorigenic and pro-fibrotic signaling.

### 3.2. VSV Shows Oncolytic Potential in CCA Cells Which Can Be Enhanced by CCA–HSC Crosstalk

**Figure 2:**
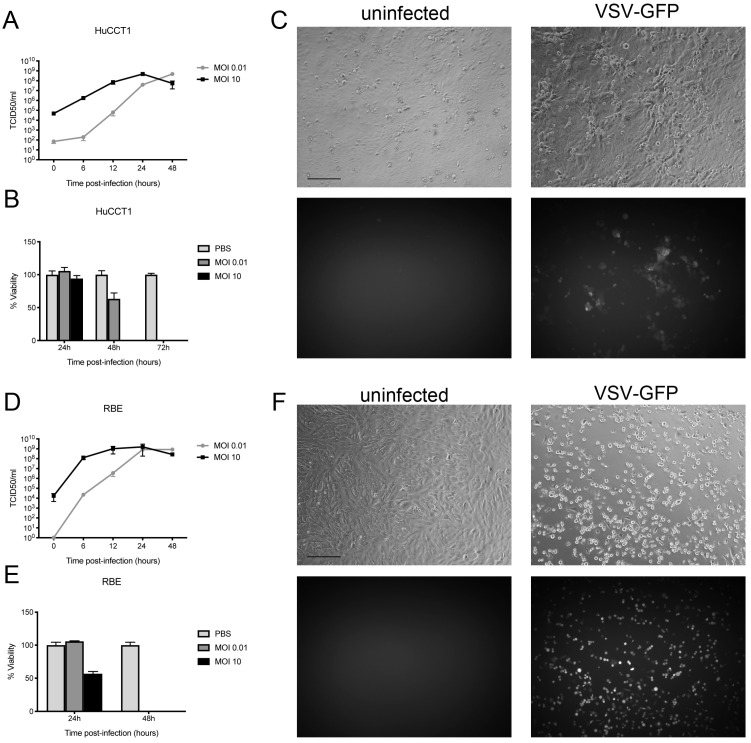
Human CCA cells are susceptible to VSV-GFP expression. Virus replication was monitored after infection of (**A**) HuCCT1 and (**D**) RBE cells at MOI 10 and 0.01 at several time points (0, 6, 12, 24, and 48 h post-infection). Viral titers were determined by TCID_50_ assay. Cell viability of (**B**) HuCCT1 was measured by MTS assay at 24, 48, and 72 h post-infection and (**E**) RBE cells at 24 and 48 h. Representative pictures of uninfected and VSV-GFP-infected (**C**) HuCCT1 and (**F**) RBE cells at 48 h post-infection are shown in bright fields (top) and fluorescence for GFP visualization (bottom). The scale bar indicates 100 µm. Mean values from three independent experiments are shown, and error bars indicate SEM.

To determine whether human CCA cells would be susceptible to VSV, HuCCT1 and RBE cells were infected with VSV-GFP at MOIs of 0.01 and 10, and viral titers and cell viability were assessed at several time points. Additionally, the extent of virus infection was monitored by fluorescence microscopy to visualize the expression of the GFP reporter gene. Expression of GFP serves as a surrogate marker of viral protein expression, as the GFP signal only becomes visible if sufficient levels of virus replication have occurred. VSV-GFP was able to replicate in both HuCCT1 and RBE cells, with maximum titers reached at 24–48 h post infection (Figure 2A,D) and vector-mediated GFP expression evident in both cell lines at 48 h (Figure 2C,F). While HuCCT1 cells were rapidly killed when infected at high MOI, infection with low MOI reduced viability to around 60% after 48 h, and complete killing was reached after 72 h (Figure 2B,C). RBE cells showed even higher susceptibility to VSV-GFP expression, as infection with MOI 10 as well as MOI 0.01 led to complete killing after 48 h (Figure 2E,F).

**Figure 3:**
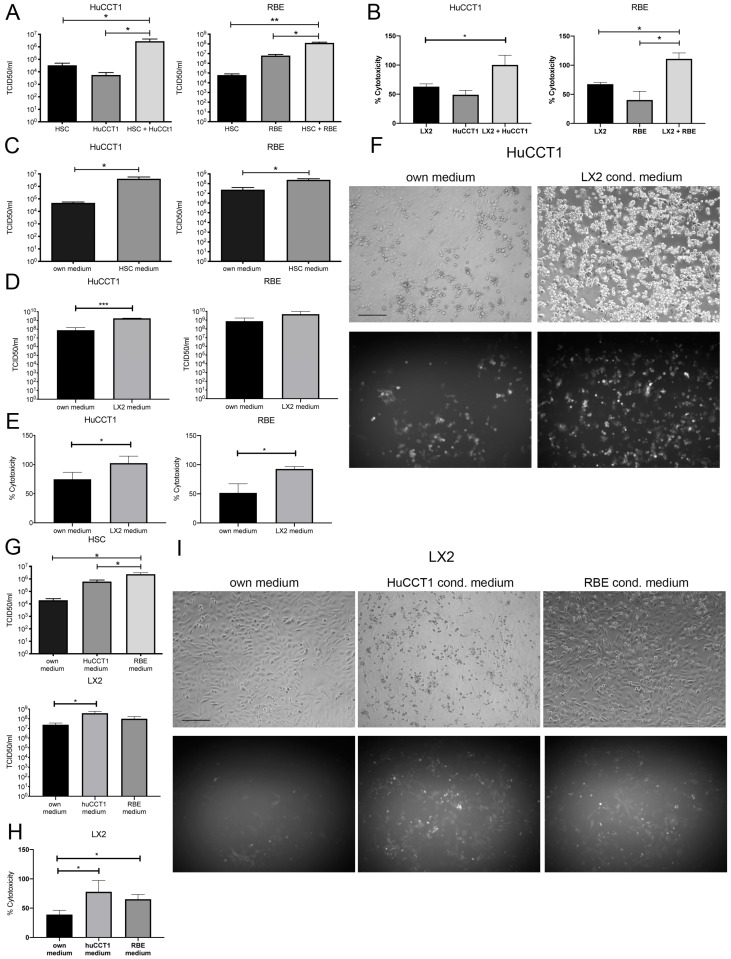
Crosstalk between CCA cells and HSCs enhances viral replication and cytotoxicity. (**A**) Viral titers were determined 24 h post-infection by TCID_50_ assay after co-culture of HuCCT1 and RBE cells with HSCs at a ratio of 1:1. (**B**) Cytotoxicity was measured by LDH release upon infection of HuCCT1 and RBE cells with VSV-GFP at MOI 0.01 for 24 h in co-culture with LX2 cells. Values were normalized to a maximum release control. TCID_50_ assay of HuCCT1 and RBE cells cultured in (**C**) primary HSC or (**D**) LX2 conditioned media were measured at 48 h post-infection. (**E**) Cytotoxicity was measured as a function of LDH release from infected HuCCT1 and RBE cells cultured in own or LX2-conditioned medium at 48 h post-infection at MOI 0.01. Values were normalized to a maximum release control. (**F**) Representative images of HuCCT1 cells infected with VSV-GFP at MOI 0.01 48 h post-infection. The scale bar indicates 100 µm. (**G**) Viral titers measured by TCID_50_ assay from VSV-GFP-infected primary HSCs and LX2 cells 48 h post-infection. (**H**) Cytotoxicity was measured by LDH release assay upon infection of LX2 cells with VSV-GFP after 48 h. Values were normalized to a maximum release control. (**I**) Representative photomicrographs of LX2 cells infected in own, HuCCT1, or RBE conditioned media with VSV-GFP at MOI 0.01 at 48 h post-infection are shown. The scale bar indicates 100 µm. Mean values from three independent experiments are shown, and error bars indicate SEM. Statistical significance was determined by Student’s *t*-test (* *p* < 0.05, ** *p* < 0.01, *** *p* < 0.001).

Direct co-culture of HuCCT1 and RBE cells with HSCs led to a 100-fold increase in VSV-GFP titers compared to either cell line cultured alone, indicating that interaction between cancer and stromal cells could enhance viral replication (Figure 3A). This was also reflected in increased cytotoxicity when HuCCT1 and RBE cells were cultured together with the HSC cell line, LX2. Cytotoxicity reached nearly 100% in co-culture, while only around 50% of cells were dead in single culture conditions at 24 h post-infection (Figure 3B). Cytotoxicity of uninfected cells was negligible.

In order to determine whether this effect was dependent on direct cell-cell contact between CCA cells and HSCs, or whether secreted factors mediate cross-talk that is responsible for enhanced viral replication, conditioned media experiments were performed. Indeed, a similar effect on viral titers was observed when HuCCT1 and RBE cells were cultured in primary HSC and LX2-conditioned medium (Figure 3C and Figure 3D, respectively) before and during infection, demonstrated by a 100-fold increase for HuCCT1 cells and a 10-fold for RBE cells compared to the culture of either cell type in their own medium. In line with that, cytotoxicity was increased from 50% (RBE cells) and 70% (HuCCT1 cells) to nearly 100% when cells were cultured in an HSC-conditioned medium (Figure 3E). This effect could also be observed microscopically (Figure 3F). Similarly, viral titers were significantly increased in primary HSCs as well as LX2 cells when cultured in HuCCT1- or RBE-conditioned medium compared to their own medium (Figure 3G), and infection of LX2 in HuCCT1- or RBE-conditioned medium led to increased cytotoxicity compared to culture in its own medium (Figure 3H). Cytotoxicity of uninfected cells in the presence of own or conditioned medium was negligible in all experiments. Microscopy at 48 h post-infection also showed increased GFP signal and cell death in conditioned media wells (Figure 3I). Taken together, these results indicated that paracrine crosstalk between CCA cells and HSCs could enhance viral replication of VSV and infection-induced cytotoxicity, which is also evident in the uni-directional communication set-up depicted in the conditioned medium experiments.

### 3.3. Crosstalk Between CCA Cells and HSCs Dampens IFN Response to Viral Infection

**Figure 4:**
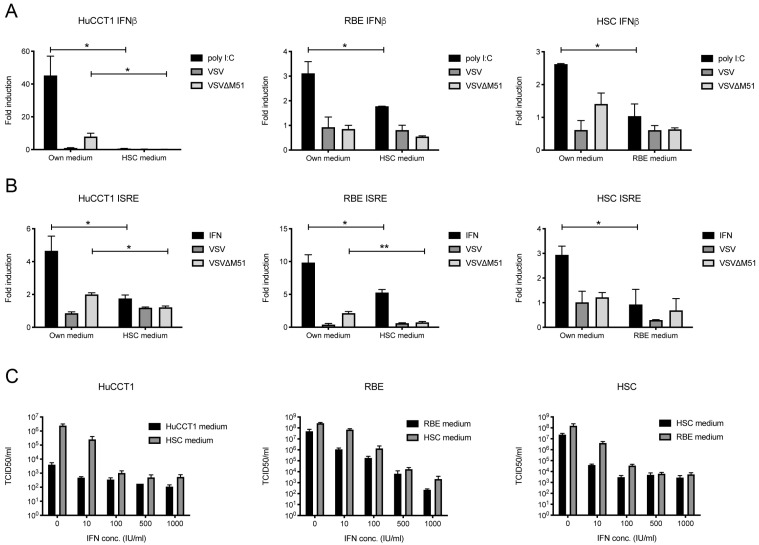
Crosstalk between CCA cells and HSCs dampens IFN signaling and response pathways. (**A**) IFNβ and (**B**) Interferon-stimulated response element (ISRE) promoter activation were measured in HuCCT1, RBE, and primary human stellate cells (HSCs) after overnight infection with VSV or VSV(MΔ51) or stimulation with poly I:C or universal type-I IFN, respectively, using luciferase reporter plasmids and the Dual-Luciferase Reporter assay. Values were normalized to control the luciferase signal and are shown as fold-induction compared to uninfected controls. (**C**) HuCCT1, RBE, and HSC cells were treated with type-I IFN overnight prior to infection with VSV at MOI 1. Viral titers were measured 18 h post-infection using TCID_50_ assay. Mean values from three independent experiments are shown, and error bars indicate SEM. Statistical significance was determined by Student’s *t*-test (* *p* < 0.05, ** *p* < 0.01).

The IFN induction and response are the key signaling pathways that are activated in response to viral infection, and their functionality strongly influences the susceptibility of cancer cells to oncolytic viruses. We, therefore, sought to determine whether changes in IFN signaling could be responsible for increased viral replication upon crosstalk of CCA cells and HSCs. To this end, human CCA cell lines, HuCCT1 and RBE, as well as primary human HSCs were transfected with reporter plasmids for the IFN induction and response pathway, and either stimulated with poly I:C or IFN, respectively, as a positive control, or infected with VSV or VSV(MΔ51). Many viruses including VSV have evolved mechanisms to dampen IFN responses to infection, making it therefore difficult to detect activation of these signaling pathways in vitro. VSV(MΔ51) carries a deletion in the viral M protein which abolishes the virus’ ability to inhibit IFN responses and was thus used additionally to analyze the activation of these pathways upon infection. HuCCT1 and RBE cells demonstrated strong upregulation of the IFN induction pathway upon stimulation with poly I:C, and this activation was highly reduced when cells were cultured in an HSC-conditioned medium. A similar effect, although less pronounced, was observed upon infection of the cells with VSV or VSV(MΔ51). Likewise, HSC cells upregulated IFN expression upon stimulation with poly I:C or infection with VSV(MΔ51), and this induction was abolished when HSC cells were cultivated in an RBE-conditioned medium (Figure 4A). Furthermore, the IFN response pathway was highly upregulated in HuCCT1 and RBE cells and in HSCs upon stimulation with type-I IFN as well as upon infection with VSV(MΔ51) when cells were cultured in their own medium, while activation of the IFN response pathway was significantly reduced when HuCCT1 and RBE cells were cultured in HSC-conditioned medium. Similarly, pathway activation was significantly reduced in HSCs stimulated with IFN when cells were cultured in an RBE-conditioned medium. When cells were treated with type-I IFN prior to VSV infection, high levels of IFN could reduce viral replication in HuCCT1, RBE, and HSCs, indicating that cells can be protected from viral infection by activation of the IFN response pathway. A similar effect was observed for CCA cells cultured in HSC-conditioned medium and HSCs cultured in RBE-conditioned medium, however, to a lesser extent. Taken together, these results indicated that communication in either direction between CCA cells and HSCs increased viral replication by inhibiting IFN activation and response pathways.

### 3.4. VSV Has Oncolytic Potential in CCA and Replicates in HSCs In Vivo

**Figure 5:**
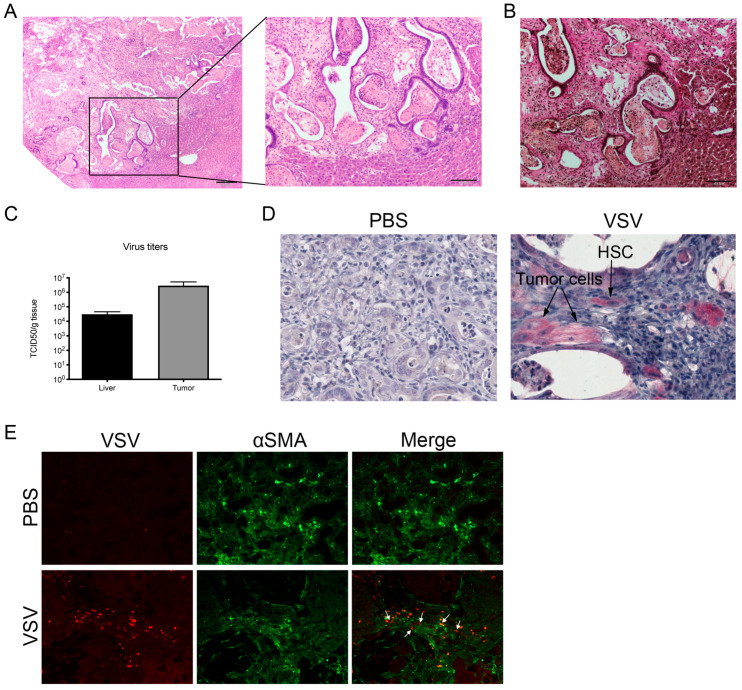
VSV replicates in tumor cells and HSCs in a rat model of CCA. Intrahepatic CCA was induced in male rats by long-term treatment with thioacetamide. Representative photomicrographs of (**A**) hematoxylin-eosin for histology analysis and (**B**) Elastica van Gieson staining for collagen (stained pink) of intrahepatic CCA sampled 24 h after treatment with PBS by hepatic arterial infusion. The scale bar indicates 100 µm. (**C**) Virus titers were measured by TCID_50_ assay from lysates of the tumor and healthy liver tissue was isolated and snap-frozen 24 h after treatment. Mean values from four individual animals are shown; error bars indicate SEM. (**D**) Representative images of immunohistochemical staining of intrahepatic CCA tumors stained for VSV-M (red) or (**E**) immunofluorescent staining of α-SMA (green) and VSV-M (red) 1 day after treatment with PBS or VSV.

To further investigate the oncolytic potential of VSV in the treatment of CCA in vivo, intrahepatic CCA was induced by thioacetamide treatment in rats. Pathological analysis of histology performed on tumor-containing liver sections indicated that, in this experimental model, rats developed poorly- to moderately-differentiated CCA surrounded by a mixed inflammation with neutrophils, macrophages, and lymphocytes. Representative structural changes were evident by hematoxylin-eosin staining (Figure 5A). The lesions were characterized by desmoplasia consistent with the clinical presentation of CCA, as depicted by Elastica van Gieson staining, which differentiates collagen fibers (pink) from normal connective tissue (Figure 5B). When rats were treated with VSV by hepatic arterial infusion, higher titers were detected in extracted CCA tissue compared to surrounding healthy liver tissue, indicating tumor-specific replication of VSV in this model (Figure 5C). Additionally, immunohistochemical staining using an antibody specific for the VSV-M protein showed that VSV could be detected in tumor cells, but not in the surrounding non-cancerous tissue. Interestingly, VSV could also be detected in adjacent intratumoral HSCs, suggesting that VSV could also replicate in HSCs in vivo (Figure 5D). Immunofluorescence staining for VSV and the HSC marker, αSMA, further confirmed VSV replication in HSCs (Figure 5E). These findings suggested that VSV could have oncolytic potential in CCA by targeting both cancer cells and surrounding cancer-associated HSCs.

### 3.5. Treatment with VSV Reduces Intratumoral Fibrosis In Vivo

**Figure 6:**
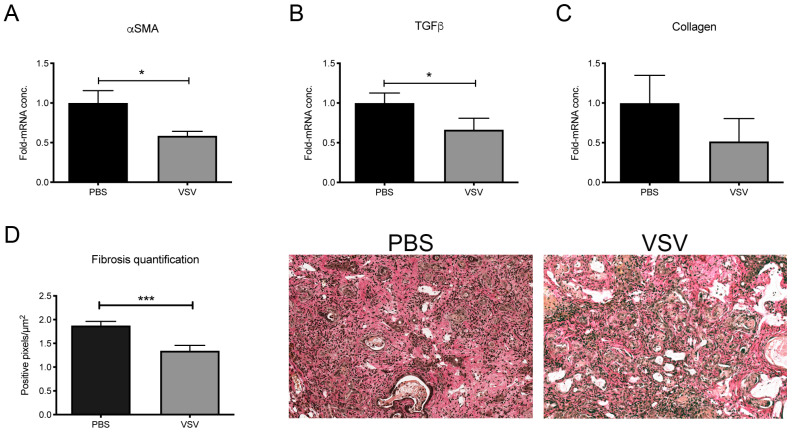
VSV reduces fibrosis in a rat model of CCA. CCA tumors were induced in rats by long-term thioacetamide treatment in drinking water, and animals were treated with PBS or VSV by intrahepatic arterial infusion. Expression of (**A**) α-SMA, (**B**) TGF-β, and (**C**) collagen was analyzed by RT-qPCR 1-day post-treatment. mRNA levels were normalized to GAPDH and are depicted as fold-change compared to PBS-treated controls. (**D**) Intratumoral fibrotic content was quantified by analysis of pink-stained collagen fibers from the Elastica van Gieson staining of VSV- or PBS-treated CCA tumors. Mean values from four animals and representative photomicrographs are shown; error bars indicate SEM. Statistical significance was determined by Student’s *t*-test (* *p* < 0.05, *** *p* < 0.001).

CCA is often associated with liver fibrosis, which not only facilitates tumor progression but can also impair drug delivery to cancer lesions. As VSV was shown to infect HSCs in vitro and in vivo in this study, we investigated the effects of OV treatment on fibrosis, which is often caused by extracellular matrix deposition by activated fibroblasts. To analyze whether VSV treatment could have an effect on fibrosis, CCA tumors were harvested from PBS- or VSV-treated rats, and fibrotic markers were measured by RT-qPCR. Tumors isolated from VSV-treated rats demonstrated a significant downregulation of αSMA, a marker for HSC activation, and TGF-β (Figure 6A and Figure 6B, respectively). Additionally, collagen levels were decreased in VSV-treated tumors compared to PBS controls (Figure 6C), and a reduction of fibrotic content could be observed histologically by quantification of collagen, which is differentially stained (pink) by Elastica van Gieson staining (Figure 6D). These results indicated that VSV treatment could represent a potent therapy for CCA, not only by directly infecting and killing tumor cells but also by therapeutically modulating tumor-associated fibrosis by targeting HSCs in the tumor stroma.

## 4. Discussion

Despite the emergence of novel targeted and immune-based therapies, stromal-rich solid tumors such as CCA remain a particular challenge due to the tumor-promoting and immune-suppressive cross-talk within the tumor microenvironment. Although clinical trials have reported some degree of therapeutic effect of gemcitabine/cisplatin treatment, and these therapeutics are used as first-line palliative treatment, the prognosis of CCA patients remains poor [36,37]. Resistance of CCA to chemotherapy has been linked to fibrosis [20,38], highlighting the importance of the TME in this cancer entity, and thus, combinatorial treatment of a fibrosis-resolving agent together with chemotherapeutic treatment could potentially improve therapy outcomes. In line with that, the combination of gemcitabine treatment with an ECM-degrading drug component was shown to inhibit tumor growth of PDAC and to prolong the survival of patients [39], further highlighting the importance of the TME in determining therapy outcomes. Targeted therapies, designed to modulate factors like abhorrent FGFR2 and isocitrate dehydrogenase (IDH), have demonstrated moderate therapeutic responses and survival prolongations in CCA [40,41,42], but further improvements are still needed. Immunotherapies, such as dendritic cell (DC) or T cell therapies or immune checkpoint inhibitors, have also been investigated for intrahepatic CCA, but these therapies have also demonstrated only limited success and only in subsets of patients, although many of these trials are still ongoing [43,44,45]. In contrast to these approaches, OVs offer the benefit of providing multiple modes of action, including direct lytic effects, immune-stimulatory activity, and as shown here, modulatory activity on the complex TME.

In this study, we showed that oncolytic VSV treatment not only infected and destroyed CCA cells, but could also target activated HSCs, the major producer of desmoplasia in these tumors, thereby reducing fibrosis. Thus, VSV might offer a multimechanistic therapeutic benefit by targeting CCA cells directly to reduce tumor burden, while additionally modulating the desmoplastic TME, thereby potentially improving therapeutic outcomes compared to chemotherapeutic treatment.

The susceptibility of cancer cells to oncolytic VSV has been strongly linked to defective type I interferon signaling. This pathway acts as a first-line defense against viral infection and is commonly altered in cancer cells since its inactivation is associated with growth advantages, while also rendering the cells more susceptible to oncolytic viruses [46,47]. We have previously demonstrated that, in addition to its tumor specificity, oncolytic VSV is also able to replicate within HSCs in an activation-specific manner within the context of hepatic fibrosis, which was directly correlated with a reduction of type I IFN signaling in activated, compared to quiescent, HSCs [31]. Interestingly, crosstalk between CAFs and various cancer cells was shown to delay secretion of IFN-β and to downregulate the expression of the viral sensor retinoic acid-inducible gene I (RIG-I), thereby sensitizing cancer cells to infection with oncolytic viruses. In line with that, conditioned media experiments in this study showed that crosstalk between HSCs and CCA cells decreased the activation of IFN induction and response pathways upon stimulation with poly I:C and IFN, respectively, or upon viral infection. However, pre-treatment with type I IFN still led to a decrease in viral titers upon infection of CCA cells and HSCs, suggesting that increased susceptibility to VSV could only be partially explained by reduced type I IFN signaling. Therefore, the specific mechanisms that might be involved in CCA-HSC crosstalk-mediated sensitizing to oncolytic virotherapy are not yet fully elucidated. Further exploration through advanced techniques, such as transcriptomic and proteomic analysis are warranted, in order to more intricately probe the molecular mechanisms driving these interactions and to identify potential therapeutic targets.

TGF-β was shown to be one of the key components regulating crosstalk between cancer cells and the TME. It can be produced by a variety of cells infiltrating the tumor stroma, including fibroblasts, leukocytes, or macrophages [48], or by the cancer cells themselves [49], and human HCC cells have been shown to upregulate TGF-β when they were co-cultured with CAFs [50]. In CCA, TGF-β expression was previously shown to be overexpressed and to promote epithelial-to-mesenchymal transition (EMT) supporting the metastatic potential of the tumors [51], and thus, to correlate with poor prognosis, metastasis, and tumor reoccurrence [52,53,54]. Furthermore, inhibition or knock-down of TGF-β reduced cell migration and the malignant potential of CCA cells, while its overexpression increased tumor volume and metastatic foci in a rat model of CCA [55]. Additionally, TGF-β has been shown to be a potent activator of stellate cells, the main precursor of CAFs in the liver [8,9,10], thereby promoting liver fibrosis [56], and these activated HSCs were shown to induce EMT and increase wound healing properties in HCC cells [21]. Similarly, TGF-β was shown to be a potent activator of pancreatic stellate cells (PSCs) which promoted EMT [57] and accelerated metastasis in this tumor type [58]. Given its cancer-promoting role in already established TME-rich tumors, inhibition of TGF-β has become a strategy of interest for the development of new cancer therapies. To that end, a selective inhibitor of the TGF-β receptor in combination with sorafenib prolonged the overall survival of HCC patients [59], while inhibition of downstream kinases of TGF-β in combination with chemotherapy improved the overall survival of PDAC patients [60,61,62]. However, the application of TGF-β inhibition for the treatment of cancer patients is challenging due to systemic effects and the complexity of cancer and fibrosis [63]. In the study presented here, VSV treatment of CCA in rats led to therapeutic modulation of fibrosis and downregulation of TGF-β and the HSC activation marker, α-SMA, concomitantly with a decrease in collagen levels. Given its specific replication in tumor cells and TME, but not in healthy tissue, VSV could therefore act as a local inhibitor of TGF-β, and thereby therapeutically modulate fibrosis, without the drawbacks of systemic alteration of TGF-β signaling. Thus, oncolytic VSV could potentially improve drug delivery of chemotherapeutic agents such as gemcitabine, making it an interesting candidate for novel combinatorial therapy approaches.

A desmoplastic TME has additionally been shown to alter immune responses directed against the tumor by different mechanisms. For instance, activated HSCs have been shown to induce M2-related genes in macrophages [21], and these M2 macrophages were shown to have tumor-promoting properties, inhibit T cells, and be negatively correlated with immunotherapy outcomes [64,65,66]. Additionally, macrophage polarization towards the M2 state is associated with poor prognosis and metastasis in CCA tumors [67,68,69], and the composition of tumor-infiltrating immune cells was shown to be predictive of patient outcomes in these tumors [70,71]. In PDAC, stiff extracellular matrix negatively influenced CD8+ T cell infiltration into the TME [72], and in breast cancer patients, the activity of tumor-infiltrating lymphocytes was shown to be influenced by collagen density [73], further highlighting the crucial role of tumor stroma in immune evasion. In general, CCA tumors are commonly described as immunologically “cold” [74,75,76], and EMT has been associated with immune evasion [77], while infiltration of these tumors with activated CD8+ T cells and NK cells mediated tumor cell killing and improved patient survival [78,79]. Thus, oncolytic VSV therapy could enhance an anti-tumor immune response by different means. By targeting activated HSCs, oncolytic virotherapy could reverse the M2 phenotype in macrophages, thereby dampening the tumor-promoting effects of this cell type. Additionally, reduction of fibrosis might lessen the stiffness of the TME and therefore facilitate infiltration of immune cells into the tumor surroundings. Ultimately, viral infection at the tumor site could also aid in converting CCA into immunologically “hot” tumors, as viral infection can induce a strong inflammatory reaction locally within the tumor. As a first step, oncolytic VSV can induce immunogenic cell death (ICD) which is crucial for the induction of an immune response against the tumor [80]. Cytokines and danger-associated molecular patterns (DAMPs) released by infected tumor cells mediate the activation of NK cells and antigen-presenting cells [81,82,83,84], which can then activate T cells, not only directed at virus-infected cells but also T cells that are specific for tumor-associated antigens, thereby mediating a strong systemic immune response [85,86,87,88].

It should be noted that, although we and others have demonstrated that VSV is a highly potent oncolytic vector, which can be safely administered at effective doses to preclinical rodent models, there are significant regulatory and safety concerns surrounding the clinical translation of VSV-based vectors. The work presented here was intended as a proof of concept to support the hypothesis that oncolytic viruses can be used to target the TME as an innovative strategy to combat stromal-rich tumors. This study did not explore the overall therapeutic effects in terms of tumor size or survival in response to VSV therapy; however, such studies would be critical in the further development of this approach for potential clinical translation. Furthermore, in order to exploit VSV as such a therapeutic vector for clinical use, we would strongly encourage exploring the application of modified variants of this vector that have demonstrated reduced off-target effects. As with any virus-based therapy, and especially with a replication-competent virus, safety needs to be at the forefront of all decisions regarding clinical translation. Patients undergoing OV treatment would need to be closely monitored, especially for cytokine responses, which can lead to adverse events. Potential risks include those associated with any natural virus infection. Furthermore, in order to further evaluate the translational potential of OV therapies like VSV for CCA, it will be necessary to utilize additional and more predictive preclinical models, such as patient-derived xenograft (PDX) models [89]. Additionally, to better enable systemic OV-based cancer therapies, cell carrier and nanoparticle-based drug delivery approaches can be explored [90,91].

## 5. Conclusions

Taken together, we have shown that oncolytic VSV has therapeutic potential in CCA by targeting both cancer cells and CAFs, thereby inducing cancer cell lysis and therapeutically modulating the dense tumor stroma. Furthermore, oncolytic virotherapy with VSV could aid in debulking the tumor mass and additionally sensitize CCA to chemo- or immunotherapy by reducing tumor-surrounding fibrosis and promoting immune cell infiltration through the inhibition of pro-tumorigenic cross-talk in the TME. Therefore, this work supports the use of oncolytic viruses as multimechanistic therapies to therapeutically modulate the TME, which makes them particularly promising for the treatment of stromal-rich cancers, such as CCA.

## Figures and Tables

**Table 1:** Primer sequences for quantitative real-time PCR.

Target	Forward Primer	Reverse Primer
αSMA (human)	5′-CAAGGGCTACCATGCCAACT-3′	5′-AGGGCCAGGACCTTGCTG-3′
TIMP-1 (human)	5′-CTTCTGGCATCCTGTTGTTG-3′	5′-AGAAGGCCGTCTGTGGGT-3′
TGF-β (human)	5′-CCCTGGACACCAACTATTGC-3′	5′-AAGTTGGCATGGTAGCCCTT-3′
GAPDH (human)	5′-GAAAGCTGTGGCGTGATG-3′	5′-GTTCAGCTCTGGGATGACCT-3′
αSMA (rat)	5′-CACCAACTGGGACGACATGG-3′	5′-CCATCTCCAGAGTCCAGCAC-3′
Collagen-I (rat)	5′-GCGGAGAGTACTGATCGACCCT-3′	5′-CCTCGGTGGACATCAGGCG-3′
TGF-β (rat)	5′-AAGAAGTCACCCG-CGTGCTA-3′	5′-TGTGTGATGTCTTTGGTTTTGTCA-3′
GAPDH (rat)	5′-CAACGACCCCTTCATTGACCTC-3′	5′-CACCAGCATCACCCCATTTG-3′

## Data Availability

The original raw data corresponding to the figures presented here are available upon request to the corresponding author.
